# Anomalous Course of the Brachial Plexus Identified During Ultrasound-Guided Brachial Plexus Nerve Block

**DOI:** 10.5152/TJAR.2022.21064

**Published:** 2022-08-01

**Authors:** Steven B. Porter, Hillary W. Garner, Bradley S. Schoch, Peter M. Murray, Christopher B. Robards, Michael J. Franco

**Affiliations:** 1 Department of Anaesthesiology and Perioperative Medicine, Mayo Clinic, Jacksonville, Florida, USA; 2 Division of Musculoskeletal Radiology, Department of Radiology, Mayo Clinic, Jacksonville, Florida, USA; 3 Department of Orthopaedic Surgery, Mayo Clinic, Jacksonville, Florida, USA; 4 Division of Plastic Surgery, Department of Surgery, Cooper University Hospital, Camden, New Jersey, USA

**Keywords:** Anomalous, brachial plexus, peripheral nerve blockade, supraclavicular, ultrasound

## Abstract

Knowledge of brachial plexus anatomy is essential when performing upper-extremity regional anaesthesia. Anomalous brachial plexus anatomy has been reported in up to 35% of patients. Variants include anomalous course of the roots anterior to, or within, the scalene musculature and abnormal separation of the cords around the subclavian artery. These anomalies have been detected with ultrasound, a valuable tool for delineating anatomy and providing imaging guidance during regional anaesthesia. We report a previously undescribed course of the brachial plexus relative to the subclavian artery within the supraclavicular fossa identified by ultrasound prior to peripheral nerve blockade.

**Main Points**

- The brachial plexus exists posterolateral to the subclavian artery in the supraclavicular fossa in most patients.
- Ultrasonography allows for real-time identification of anomalous brachial plexus elements.
- We report an anteromedial orientation of the brachial plexus in the supraclavicular fossa as identified on ultrasonography.

## Introduction

Over a century has passed since Halstead and Hall first used regional anaesthesia for extremity surgery by injecting 4% cocaine around the brachial plexus and tibial nerve.^1^ Although there have been steady improvements in regional anaesthesia since these earliest applications, the last 2 decades have shown considerable advancements in safety and efficacy. These enhancements have been due, in part, to the incorporation of ultrasound (US) guidance.^2,3^ The advent of US guidance has allowed regional anaesthesiologist to become more adept at identifying normal and anomalous neural, vascular, and muscular anatomy throughout the body, including variant relationships between the brachial plexus and subclavian artery. We describe a previously unreported anomalous arrangement of the brachial plexus with the subclavian artery detected with US, highlighting its role in delineating important anatomy prior to regional anaesthesia.

### Case Presentation

**Figure 1.**
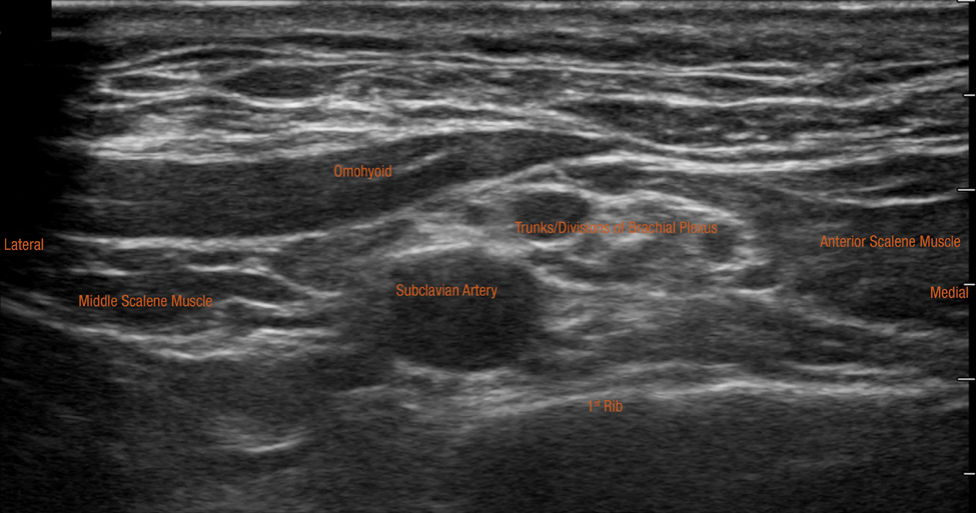
Anomalous orientation of the brachial plexus at the level of the supraclavicular fossa.

**Figure 2.**
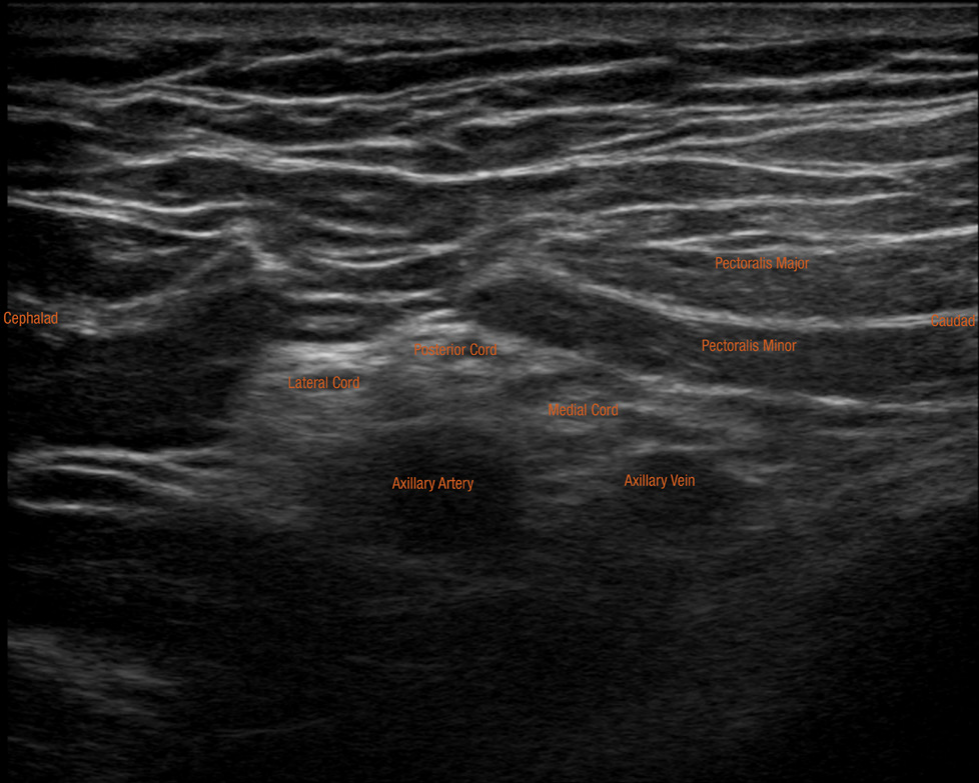
Anomalous orientation of the brachial plexus at the level of the infraclavicular fossa.

**Figure 3.**
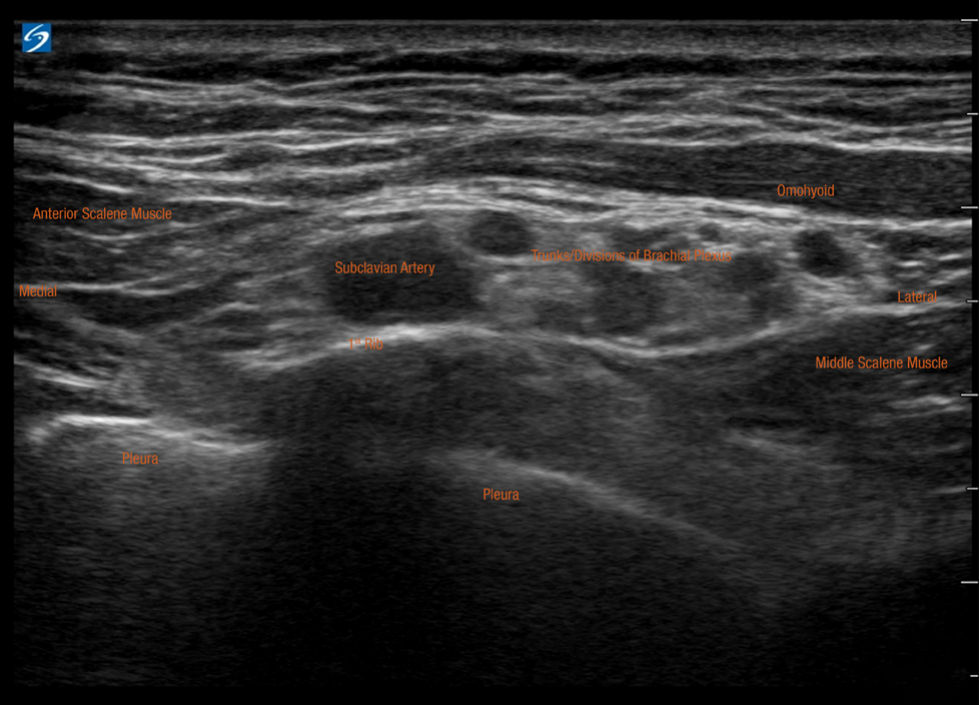
Normal orientation of the brachial plexus at the level of the supraclavicular fossa.

A 71-year-old man presented to the Department of Orthopaedic Surgery outpatient clinic for revision rotator cuff repair surgery. He demonstrated full functional range of motion, with preserved strength and pain with resisted overhead motion. His neurologic examination demonstrated normal and equivalent sensation throughout the upper extremities. After physical therapy was unsuccessful, he was offered surgery. The postoperative pain control plan included preoperative placement of an interscalene brachial plexus nerve block catheter. During routine US evaluation of the right supraclavicular fossa using a 15-6 MHz linear probe (X-Porte, Sonosite, Fujifilm), an anomalous course of the brachial plexus was discovered. Instead of the anticipated position of the brachial plexus divisions posterolateral to the subclavian artery, the divisions were found coursing anteromedial to the subclavian artery (Figure 1). Videography of the region better demonstrates the orientation of the brachial plexus, the roots of which coalesce to become divisions around the subclavian artery (Video 1). Following recognition of this anomaly, the plexus was tracked more distally along the axillary artery in the infraclavicular space (Figure 2). In this location, the usual orientation of the cords of the brachial plexus is medial, lateral, and posterior to the axillary artery, anatomic positions by which these individual cords are named. In our patient, however, all 3 cords were positioned anterior to the axillary artery. Upon US examination of the contralateral nonoperative extremity, we found the expected orientation and course of the brachial plexus (Figure 3). Of note, the patient denied any prior history of trauma to his operative arm, shoulder, or neck. We decided to place a brachial plexus catheter using a more proximal interscalene approach, where his anatomy appeared normal. The block provided adequate postoperative analgesia, and the patient’s postoperative inpatient and outpatient course was uneventful. Review of prior electromyography of the ipsilateral arm and magnetic resonance imaging of his cervical spine revealed no information to suggest anomalous orientation or conduction abnormalities of his peripheral nerves.

## Discussion

Ultrasound guidance has become an essential part of the standard of care for administering regional blocks. In a randomized trial of 160 patients, Kapral et al^2^ found that US guidance was substantially superior to nerve stimulation for interscalene blocks. Additional research in regional anaesthesia has shown the particular value of US guidance in cases of anatomic variants.^3,4^ Dolan^5^ described anomalous anatomy in an 83-year-old woman undergoing a planned interscalene plexus block for subacromial decompression of the shoulder. During the initial scan, roots C5 and C6 were located medial to the anterior scalene muscle. With the use of US, the block was safely and effectively administered, and anaesthesia was achieved in the C5-C6 distribution.^5^ Chin et al^6^ describe a case of anomalous plexus anatomy discovered during a US-guided supraclavicular block. Upon scanning the plexus in the supraclavicular fossa, the superior trunk was found to travel medial to the subclavian artery, whereas the middle and inferior trunks were in their expected locations. Upon further inspection, they found C5 and C6 travelled medial to the anterior scalene muscle. They achieved adequate anaesthesia using a modified in-plane approach at the level of the first rib, with 2 separate boluses injected.^6^

Although previous authors have described US-detected anomalous brachial plexus anatomy, both at the level of the anterior scalene and about the supraclavicular artery,^4,5^ the unusual anatomy of the brachial plexus divisions positioned medial to the subclavian artery in the supraclavicular fossa in our patient has not been previously demonstrated with US. In over 35 years of combined experience with US-guided peripheral nerve blockade, our anaesthesia team has never encountered this orientation of the brachial plexus.

Ultrasound-guided supraclavicular block was first described by Chan et al.^7^ In their description, the US probe was placed in the supraclavicular fossa parallel to the clavicle to identify the brachial plexus and subclavian artery. They used an in-plane lateral to medial needle advancement approach, which is ideal when the brachial plexus is located in its usual position because the needle is able to encounter the brachial plexus before the possibility of coming in contact with the artery. Unfortunately, in our patient, the subclavian artery would need to be traversed (and hopefully avoided) en route to the medially located brachial plexus. Singh et al^8^ described significant morbidity associated with injury to the subclavian artery, even when attempting this block in patients with a suspected normal anatomic relationship, although US guidance was not used. Variations in the take-off of the suprascapular nerve, the separation of the superior trunk, and the course of the C5 and C6 nerve roots from the neural foraminae are all variable and are best localized with real-time US. Given our patient’s anatomy, it would have been prudent to employ a medial to lateral needle advancement approach to minimize the chance of vascular injury if a supraclavicular block had been chosen. We elected to move the US probe more proximally to perform an interscalene brachial plexus block where the patient’s anatomy appeared normal.

## Conclusion

Our case provides additional evidence of the wide variations in brachial plexus anatomy, even in an individual whose contralateral, nonoperative brachial plexus was in its expected position. Although the value of US is well established, highlighting the benefits of real-time dynamic US in unique situations of regional anaesthesia delivery (e.g., anomalous anatomy) is important. Further study of US-guided peripheral blockade is paramount to advancing its safety and efficacy.
